# Long-Term Outcome of Vitrectomy with Suitable Internal Limiting Membrane Peeling and Air Tamponade for Highly Myopic Foveoschisis-Associated Lamellar Macular Hole

**DOI:** 10.1155/2020/2074037

**Published:** 2020-02-21

**Authors:** Jia-lin Wang, Yan-ling Wang

**Affiliations:** Department of Ophthalmology, Beijing Friendship Hospital, Capital Medical University, No. 95 Yongan Road, Xicheng District, Beijing, China

## Abstract

**Purpose:**

To investigate the outcome of pars plana vitrectomy (PPV) with suitable internal limiting membrane peeling (ILM) and air tamponade for patients with highly myopic foveoschisis-associated lamellar macular hole (MH).

**Methods:**

This retrospective interventional case series included 11 patients with highly myopic foveoschisis-associated lamellar MH who underwent PPV and indocyanine green-aided ILM peeling up to the temporal vascular arcades. Following air tamponade after surgery, all patients were instructed to maintain a face-down position. The patients were followed up for over 1 year and evaluated for MH closure and the best-corrected visual acuity before and after surgery.

**Results:**

The mean ± standard deviation values of patient age, axial length, and follow-up duration were 67.82 ± 6.54 years, 29.21 ± 1.95 mm, and 24.27 ± 8.11 months, respectively. After surgery, the lamellar MH closed in all eyes, and 10 eyes showed vision improvement at the 1-month, 3-month, and final follow-up evaluations. One patient showed decreased vision at 2 years after surgery, with patchy chorioretinal atrophy in the macular region. Myopic foveoschisis showed resolution in three eyes and alleviation in eight. Ten patients underwent cataract surgery during PPV.

**Conclusion:**

Extension of ILM peeling up to the temporal vascular arcades and air tamponade after PPV may improve the visual function and rate of MH closure for patients with highly myopic foveoschisis-associated lamellar MH.

## 1. Introduction

High myopia is characterized by progressive axial elongation that confers an increased risk of specific macular diseases, such as foveoschisis and macular hole (MH), with or without associated retinal detachment [1]. Myopic foveoschisis, first described by Takano and Kishi [2], was identified after the development of optical coherence tomography (OCT). Myopic foveoschisis may occur in patients with high myopia associated with posterior staphyloma, chorioretinal atrophy, and myopic degeneration. However, it is more conceivable that myopic foveoschisis results from the progressive elongation of the myopic globe at a younger age, which contributes to the development of abnormal vitreous traction and subsequent anomalous posterior vitreous detachment. This is further complicated by splitting of the neural retina and detachment of the photoreceptors from the retinal pigment epithelium [3].

A treatment option for myopic foveoschisis is surgery, although the timing of surgery remains controversial. Most surgeons decide on surgical intervention if there is a decline in visual acuity, distorted vision, or macular detachment and other complications associated with MH. However, the visual and anatomical outcomes are usually poorer for highly myopic MH than they are for idiopathic MH [4]. Furthermore, there is great disparity among the surgical results obtained after treatment of highly myopic MH in the literature [5–7]. Based on the existing reports, we discovered that the extent of internal limiting membrane (ILM) peeling is crucial for the MH closure rate in patients with high myopia. Specifically, failure in completely removing the force of traction [8, 9] may be responsible for a poor MH closure rate, while a larger extent of ILM peeling (beyond the vascular arcades to the nasal side of the optic disc) can improve the MH closure rate. The potential disadvantages of extensive ILM peeling include a decrease in retinal sensitivity, formation of iatrogenic holes, and phototoxicity arising from longer surgical durations, among others [10]. Therefore, in this study, we investigated the outcomes of patients with highly myopic foveoschisis-associated lamellar MH who were treated with pars plana vitrectomy (PPV) with suitable internal limiting membrane peeling and air tamponade.

## 2. Materials and Methods

The study was performed in accordance with the World Medical Association's Declaration of Helsinki. We retrospectively reviewed the records of 11 patients who underwent vitrectomy and suitable ILM peeling due to myopic foveoschisis-associated lamellar MH. The inclusion criteria were as follows: patients with high myopia eyes (spherical refractive error > −6.0 D and/or axial length > 26 mm) diagnosed with foveoschisis-associated lamellar MH using optical coherence tomography (OCT), and those who were available for the scheduled follow-up evaluations. Exclusion criteria included patients with idiopathic or traumatic MH, proliferative vitreoretinopathy, and retinal detachment and patients who were unavailable for follow-up examinations. Eleven eyes of 11 patients were recruited from October 2015 to January 2018 at Beijing Friendship Hospital, Capital Medical University (Beijing, China).

Each patient's ophthalmic history was obtained, and a complete ophthalmic examination was performed before the procedure, including the Snellen best-corrected visual acuity (BCVA), noncontact tonometry, slit-lamp examination, fundus color photography, axial length measurement by optical biometry (IOL Master; Carl Zeiss Meditec, Jena, Germany), and assessment of visual significance of lens status. OCT (Heidelberg Engineering, GmbH, Heidelberg, Germany) was performed for each patient before surgery and at the 1-month, 3-month, 6-month, 1-year, and final follow-up evaluations.

All procedures were performed under retrobulbar anesthesia by an experienced surgeon (YLW). Conventional three-port vitrectomy was performed using a 23-gauge system. Phacoemulsification and intraocular lens implantation were performed with vitrectomy. To facilitate visualization of the vitreous cortex, 0.1 mL triamcinolone acetonide (concentration: 0.1 mL/4 mg) was injected into the vitreous cavity during surgery. Manual posterior vitreous detachment (if absent) was induced using the aspiration function of the vitrectomy probe. After removal of the vitreous cortex, the ILM was stained with indocyanine green (0.5%) with a 1 mL syringe. After 1 min, excessive indocyanine green present in the vitreous cavity was aspirated with a vitrectomy probe. The preretinal membrane was excised with forceps, if present. ILM peeling was initiated by pinching the ILM up to the temporal vascular arcades using end-gripping forceps. The ILM was peeled in a centripetal direction to avoid the formation of an MH. ILM peeling should extend up to the temporal vascular arcades. Fluid-air exchanges were performed. Finally, air tamponade of the vitreous cavity was performed. After surgery, all patients maintained the face-down position for at least 12 h/day; this was continued for almost 1 week. Follow-up examinations were scheduled at 1 week, 1 month, 3 months, 6 months, and 1 year after surgery. Some patients were followed up for 2 or 3 years after surgery.

We checked OCT images for the absence of a neurosensory defect over the fovea (MH closure) and retinoschisis in the central macular region. The same examiner (JLW) judged complete closure of the MH and anatomical resolution of retinoschisis. The BCVA results obtained with the Snellen chart were converted to logarithm of the minimum angle of resolution (logMAR) units for statistical analysis.

Statistical analysis was performed using SPSS 22.0 statistical software. Paired *t*-tests and Wilcoxon matched-pair signed-rank tests were used as appropriate. All continuous data are expressed as the mean ± standard deviation. Differences were considered statistically significant at *p* < 0.05.

## 3. Results

The preoperative and postoperative basic characteristics of the 11 patients are summarized in Table 1. Eleven eyes of 11 patients (three men and eight women; mean age: 67.82 ± 6.54 years, range: 58 to 80 years) were included in our study. The mean axial length was 29.21 ± 1.95 mm (range: 26.98 to 32.81 mm). The mean preoperative logMAR BCVA was 0.943 ± 0.599. One eye was pseudophakic, and cataract surgery was not performed in that eye. Cataract extraction and intraocular lens implantation were performed for 10 eyes, in addition to the lamellar MH surgery. The mean follow-up duration was 24.27 ± 8.11 months.

The mean BCVA showed gradual improvement during the first 3 months of follow up, after which it tended to stabilize. As gas remains in the vitreous cavity after surgery, the BCVA obtained at the 3-month follow-up evaluation was used for statistical analysis. The mean preoperative logMAR BCVA was 0.943 ± 0.599 (Snellen equivalent: counting fingers, −20/50), and the mean postoperative logMAR BCVA was 0.312 ± 0.261 (Snellen equivalent: 20/200–20/25). When compared with the preoperative BCVA, the changes in the mean BCVA obtained at the 3-month follow-up evaluation were statistically significant (*p* < 0.05). Ten eyes (90.91%) showed both subjective and objective improvements in visual acuity after surgery. The patient in Case 3 maintained the same BCVA 3 months after surgery, and the eye showed a decrease in visual acuity (20/1000) 2 years after surgery. Other patients maintained the same BCVA at the final follow-up time as that measured at the 3-month follow-up examination.

**Figure 1:**
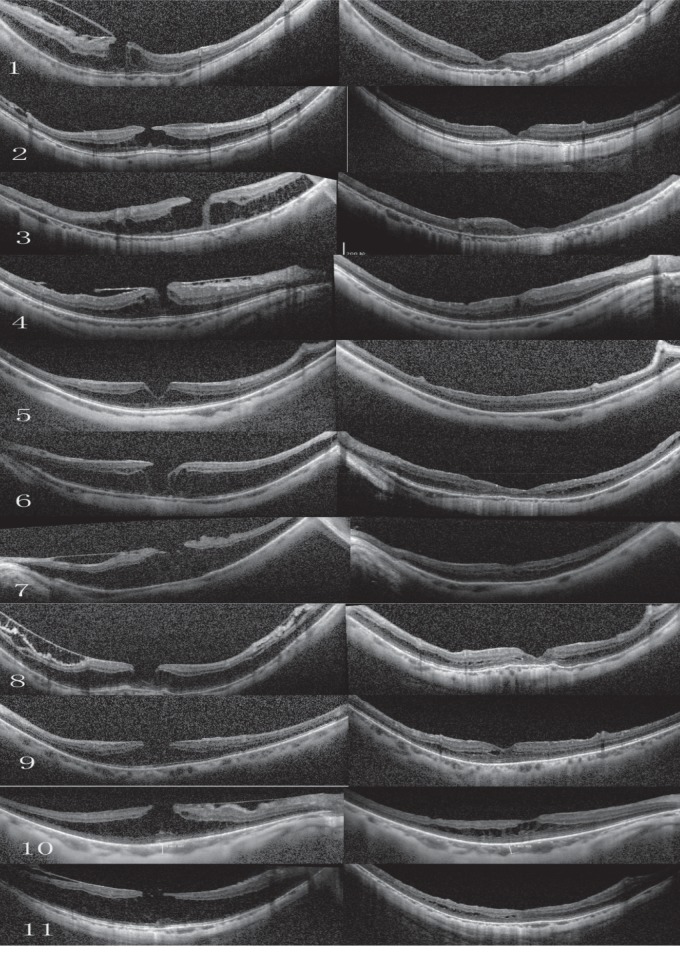
Preoperative and postoperative optical coherence tomography (OCT) scans for the 11 patients who underwent vitrectomy and suitable internal limiting membrane (ILM) peeling. The scans are shown as pairs (left, preoperative OCT; right, postoperative OCT) in chronological order.

**Figure 2:**
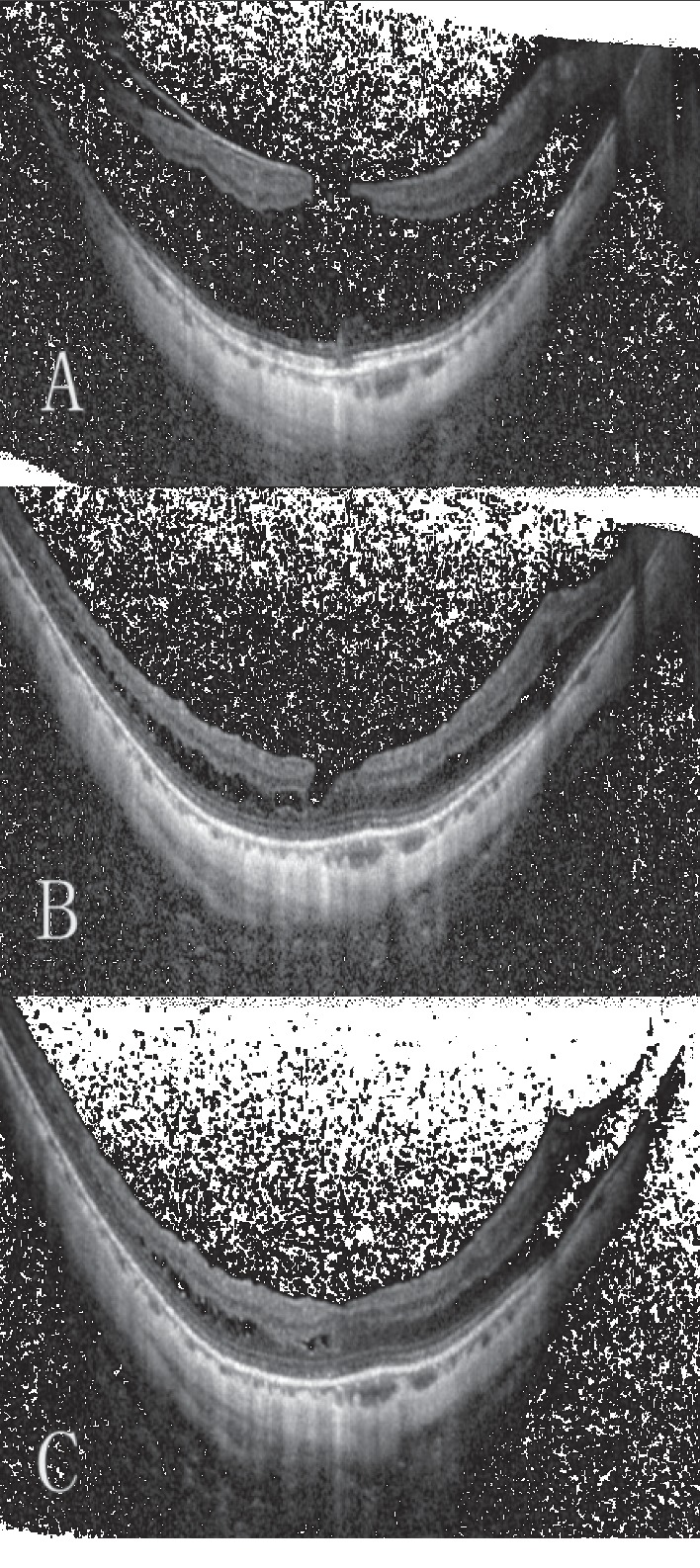
Preoperative and postoperative images for a representative case of highly myopic foveoschisis-associated lamellar macular hole treated by vitrectomy with suitable internal limiting membrane peeling and air tamponade (Case 11). (a) Preoperative optical coherence tomography (OCT) images reveal foveoschisis and lamellar macular hole. The Snellen vision acuity was 20/60. (b) OCT image obtained at the 3-month follow-up evaluation shows that the macular hole has closed and the retinoschisis has reduced. The Snellen vision acuity was 20/25. (c) OCT image obtained at the 1-year follow-up evaluation shows a closed macular hole with further reduction in the retinoschisis. The final Snellen vision acuity was 20/25.

All eyes exhibited posterior scleral staphyloma and foveoschisis. Silicone oil tamponade after vitrectomy was performed for one eye. The others underwent air tamponade after vitrectomy. MH closure was achieved and confirmed by OCT in 11 patients (100%). Retinoschisis resolved in four eyes. Some patients showed mild extrafoveal schisis, while two patients showed mild foveoschisis (Figures 1 and 2). We did not observe any serious intraoperative or postoperative complications. The patient in Case 1 had a history of cataract surgery 5 years previously, while the other patients had a history of cataract surgery combined with vitrectomy.

## 4. Discussion

**Figure 3:**
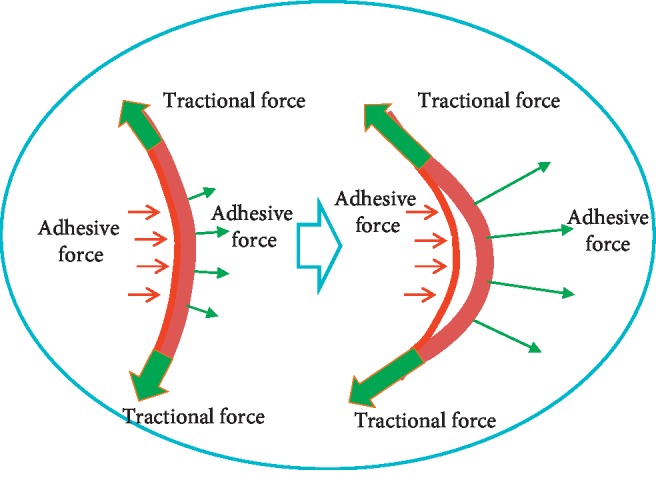
Possible mechanism of force in patients with myopic foveoschisis. The image on the left belongs to a nonmyopic patient. The image on the right belongs to a patient with myopic foveoschisis.

In the present study, the extent of ILM peeling to the temporal vascular arcade was a crucial part of the procedure. As other studies have reported, limiting the range of ILM peeling within the vascular arcade may lead to a poor MH reclosure rate [8, 9, 11] due to a failure in completely removing tractional force. We found that the ILM in patients with myopic foveoschisis was significantly different from that in patients with idiopathic MH. Bando et al. [12] discovered that the distribution of collagen fibers and cellular debris on the inner surface of the ILM in patients with myopic foveoschisis and high myopia indicated poor ILM elasticity. Thus, we infer that the tractional force and adhesive force at the posterior retina increased along with the increase in the axial length. Due to the poor elasticity of the ILM in patients with myopic foveoschisis, small distraction deformation and the action of the adhesive forces before and after, together with the separation, resulted in traction (Figure 3). We peeled off the ILM up to the temporal vascular arcade to eliminate the tractional force to a greater extent. The MH exhibited closure in all patients, with stable results during the long-term follow-up. In order to prevent the development of an MH, we peeled the ILM in a centripetal direction, in patients with myopic foveoschisis-associated lamellar MH.

Recently, a meta-analysis [13] reported that gas tamponade might cause more complications compared to vitrectomy without tamponade, although no significant differences were found in the outcomes of visual acuity or in the resolution of myopic foveoschisis. Another report [14] demonstrated a high myopic foveoschisis resolution rate (88%) with intraocular air tamponade. Maintaining the face-down position for a short period after surgery is useful for reducing the patients' visual and postural discomfort. Considering the potential risks of silicone oil tamponade and the necessity of secondary removal, air tamponade is preferred for myopic foveoschisis-associated lamellar MH. Only one patient (Case 3) underwent tamponade with silicone oil. The preoperative BCVA (20/200) was maintained at 3 months after surgery, and the eye showed a decrease in visual acuity (20/1000) at 2 years after surgery. Anatomical success was achieved with MH closure and resolution of foveoschisis. Unfortunately, patchy chorioretinal atrophy was detected in the macula at 2 years after surgery. Nevertheless, we observed that air tamponade did not cause these issues in other patients.

The timing of surgery is important. In the current study, all patients with high myopia who needed surgery had inner segment/outer segment junction disruptions before surgery [15]. Lai et al.'s study [16] also reported that patients with lamellar MH showed improved vision after surgery. Therefore, we chose patients with high myopia and foveoschisis-associated lamellar MH and blurred vision for our study. Except for the patient with patchy atrophy in the macular area, all patients demonstrated an improved visual acuity after surgery.

The limitations of our study include its retrospective nature, relatively small sample size, and no comparison with a control group. Except for Case 1, where cataract surgery was performed before 5 years, all cases received cataract surgery along with vitrectomy. However, their lens opacity did not interfere with vision. We were also able to visualize the patients' fundus. Considering the rapid development of cataract after vitrectomy and the difficulty in performing cataract surgery after vitrectomy for the same eye, we performed both procedures together.

To date, few articles on patients with high myopia and foveoschisis-associated lamellar MH have been published. Herein, we found that patients with high myopia and foveoschisis-associated lamellar MH closure who were treated with vitrectomy and ILM peeling showed BCVA improvements. There were no serious complications during the long-term follow-up. Collectively, our findings suggest that PPV, suitable ILM peeling, and air tamponade should be performed to achieve high anatomical success rates and visual acuity improvements in patients with high myopia and foveoschisis-associated lamellar MH closure. Moreover, in order to prevent MH development, ILM peeling should be performed in a centripetal direction. We believe that the diameter of ILM peeling is important. Further randomized case-control studies with larger sample sizes are required to confirm the utility of these techniques.

## Figures and Tables

**Table 1:** Basic characteristics of patients (*n* = 11) with highly myopic foveoschisis-associated lamellar macular hole.

No.	Sex	Age year	Eye	AXL°mm	Posterior staphyloma	Cataract	Gas used	Follow-up	Preop BCVA logMAR (Snellen)	Final BCVA logMAR (Snellen)	BCVA improvement
1	M	65	OD	32.81	Present	Pre-5°Y	Air	46	0.398 (20/50)	0.301 (20/40)	Yes
2	M	67	OD	29.95	Present	Yes	15%C3F8	42	1 (20/200)	0.1 (20/25)	Yes
3	F	69	OS	29.06	Present	Yes	Silicone	32	1 (20/200)	1 (20/200)	No
4	M	63	OD	28.06	Present	Yes	Air	31	0.398 (20/50)	0.176 (20/30)	Yes
5	F	76	OD	28.05	Present	Yes	Air	25	0.699 (20/100)	0.1 (20/25)	Yes
6	F	69	OS	28.41	Present	Yes	Air	25	1 (20/200)	0.301 (20/40)	Yes
7	F	80	OS	26.98	Present	Yes	Air	25	1 (20/200)	0.481 (20/60)	Yes
8	F	65	OD	30.46	Present	Yes	Air	25	0.699 (20/100)	0.176 (20/30)	Yes
9	F	73	OS	27.55	Present	Yes	Air	22	0.398 (20/50)	0.301 (20/40)	Yes
10	F	58	OD	27.69	Present	Yes	Air	19	2.3 (CF/30 cm)	0.398 (20/50)	Yes
11	F	61	OD	32.28	Present	Yes	Air	15	0.481 (20/60)	0.1 (20/25)	Yes

## Data Availability

All relevant data are included within the article.
